# *Sulfolobus acidocaldarius* UDG Can Remove dU from the RNA Backbone: Insight into the Specific Recognition of Uracil Linked with Deoxyribose

**DOI:** 10.3390/genes8010038

**Published:** 2017-01-18

**Authors:** Gang-Shun Yi, Wei-Wei Wang, Wei-Guo Cao, Feng-Ping Wang, Xi-Peng Liu

**Affiliations:** 1 State Key Laboratory of Microbial Metabolism, School of Life Sciences & Biotechnology, Shanghai Jiao Tong University, Shanghai 200240, China; 13166228531@163.com (G.-S.Y.); www1037554814@sjtu.edu.cn (W.-W.W.); fengpingw@sjtu.edu.cn (F.-P.W.); 2 Department of Genetics and Biochemistry, Clemson University, Clemson, SC 29634, USA; wgc@clemson.edu; 3 State Key Laboratory of Ocean Engineering, School of Naval Architecture, Ocean and Civil Engineering, Shanghai Jiao Tong University, Shanghai 200240, China

**Keywords:** *S. acidocaldarius*, uracil-DNA glycosylase, [4Fe-4S] cluster, RNA backbone

## Abstract

*Sulfolobus acidocaldarius* encodes family 4 and 5 uracil-DNA glycosylase (UDG). Two recombinant *S. acidocaldarius* UDGs (SacUDG) were prepared and biochemically characterized using oligonucleotides carrying a deaminated base. Both SacUDGs can remove deoxyuracil (dU) base from both double-stranded DNA and single-stranded DNA. Interestingly, they can remove U linked with deoxyribose from single-stranded RNA backbone, suggesting that the riboses on the backbone have less effect on the recognition of dU and hydrolysis of the C-N glycosidic bond. However, the removal of rU from DNA backbone is inefficient, suggesting strong steric hindrance comes from the 2′ hydroxyl of ribose linked to uracil. Both SacUDGs cannot remove 2,2′-anhydro uridine, hypoxanthine, and 7-deazaxanthine from single-stranded DNA and single-stranded DNA. Compared with the family 2 MUG, other family UDGs have an extra N-terminal structure consisting of about 50 residues. Removal of the 46 N-terminal residues of family 5 SacUDG resulted in only a 40% decrease in activity, indicating that the [4Fe-4S] cluster and truncated secondary structure are not the key elements in hydrolyzing the glycosidic bond. Combining our biochemical and structural results with those of other groups, we discussed the UDGs’ catalytic mechanism and the possible repair reactions of deaminated bases in prokaryotes.

## 1. Introduction

Some DNA damage in the genome is harmful to the cell if not repaired. Deoxyuracil (dU) is a kind of DNA damage and exists in the form of a U/G or U/A base pair. U/G mismatch results from the hydrolysis deamination of the exocyclic amino group of cytosine in DNA, and a U/A base pair is generated via the misincorporation of dUMP into the DNA opposite base A during replication. If not repaired, the G:U mismatched base pair will generate a permanent G:C to A:T transition mutation after replication. In addition to dU damage, hydrolysis deamination of the purines adenine and guanine inflicts damage on hypoxanthine and xanthine, respectively [1]. The rate constants for hydrolysis deamination of bases in DNA at elevated temperatures are several orders of magnitude higher than those at more moderate temperatures [2]. Hence, hyperthermophiles face a serious high-temperature threat because of their special living environment. As such, several strategies are developed to address such damages. For example, dUTPase can hydrolyze harmful dUTP to avoid the incorporation of dUMP [3,4]. Second, several enzymes are responsible for eliminating dU damage [5,6,7,8]. Third, family B DNA polymerase tightly binds a dU base in the template strand and stagnates ahead of dU damage, thus preventing the generation of a U:A base pair [9]. Therefore, these proteins play a major role in counteracting dU damage.

Among these proteins, uracil DNA glycosylase (UDG) is found in most eukaryotes and prokaryotes. UDG removes uracil lesions in DNA by hydrolyzing the glycosidic bond between uracil and deoxyribose. Generally, organisms have more than one type of UDG [10]. At present, the UDG superfamily is classified into seven families based on their conserved motifs, substrate specificity, and structural similarity [11,12]. Family 1 UDGs (also called UNGs), as represented by *Escherichia coli*, human, and herpes simplex virus 1 UDGs, are the first characterized group of enzymes [5,13]. Family 2 is composed of human thymine DNA glycosylase (TDG), *E. coli* mismatch-specific UDG (MUG), and fission yeast (*Schizosaccharomyces pombe*) TDG [14]. Family 3 enzymes include various eukaryotic single-strand-selective monofunctional UDGs (SMUG1) and ones in few bacteria [15]. Family 4 UDGs are a group of prokaryotic [4Fe-4S] cluster-containing enzymes that act on both single-stranded and double-stranded uracil-containing DNA [16]. Family 5 UDGs, which also contain typical [4Fe-4S] clusters, show higher sequence similarity with family 4, and can remove the uracil and other damaged bases from double-stranded DNA [6]. Notably, family 4 and 5 are the first UDGs found in thermophiles [6,16], but they are also widespread in mesophilic and psychrophilic prokaryotes [10]. In comparison with family 4, family 5 only exists in minor prokaryotes. Family 6 is a DNA glycosylase specific to hypoxanthine damage (HDG family) [12]. HDG and MUG have a comparable peptide length but low sequence similarity. Recently, the family 7 UDG was identified in prokaryotes [11]. Unique from the rest, family 7 UDG does not exhibit any uracil excision activity, but has a very strong uracil-DNA binding capability that is even resistant to urea [11].

Enzymes in the UDG superfamily adopt the typical structure of a four-stranded β-sheet surrounded by α-helices, with two conserved active-site motifs of motifs A and B [5,17]. Motif A is responsible for activating the catalytic water molecule via a conserved general acid. Motif B can interact with the minor groove of double-stranded DNA once the dU base is flipped out into the active site and stabilizes the protein-DNA complex [18].

Iron-sulfur (Fe-S) cluster is a key redox molecule that exists in many proteins, including some enzymes for DNA metabolism. The [4Fe-4S] clusters have been observed in several kinds of DNA repair enzymes, including DNA glycosylases UDGIV/V [6,16], HhH TDG [19], endonuclease III [20], mutY [21], and DNA helicase XPD [22]. However, the Fe-S clusters in DNA repair enzymes seldom involve electron transfer like those in oxyreductase. The changes of Cys to Ser of UDGIV from *Pyrococcus furiosus* and *Archaeoglobus fulgidus* only result in a 40% decrease in dU removal activity [23,24], confirming that the main function of Fe-S clusters is not in binding and catalyzing the hydrolysis of the N–C glycosidic bond [25]. In contrast, some DNA glycosylases, such as endonuclease III, mutY, and HhH TDG, have a [4Fe-4S] cluster that is responsible for directly binding the substrate DNA during hydrolysis of the glycosidic bond [19,20,21]. In the case of mutY, disruption of Fe-S clusters leads to the inactivation of glycosylase and AP lyase [26].

Furthermore, disruption of the *ung* gene results in an increased mutation rate of G:C to A:T in *E. coli* [27,28]. Previous studies show that the mutation rate of G:C to A:T is also significantly increased following the order: *udg*IV and *udg*V double mutant > *udg*IV mutant > *udg*V mutant in *Thermus thermophilus* [29]. These results indicate that UDG families 1, 4, and 5 all function in vivo to remove dU in DNA.

**Figure 1:**
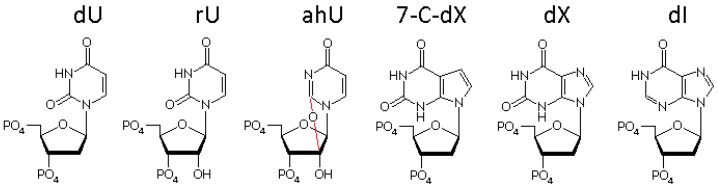
The structures of the main deaminated bases.

*Sulfolobus acidocaldarius* is a strictly aerobic and acidophilic archaea that grows at temperatures ranging from 65 to 85 °C and pH 2.0 to 4.5 [30]. Two potential UDGs (family 4 Saci_0159 and a truncated family 5 Saci_1756) are encoded in the *S. acidocaldarius* genome. To characterize the catalytic function of N-terminal secondary structures of UDGV and specific recognition of deaminated bases linked to deoxyribose or ribose via glycosidic bond (Figure 1), we expressed and purified the recombinant UDGs. Our results showed that two *S. acidocaldarius* UDGs can remove dU residues from both single- and double-stranded DNA. The deletion of 46 N-terminal residues of full-length SacUDGV does not abolish the enzyme activity, rather, only a loss of 40% activity, indicating that the Fe-S cluster is not a key element in hydrolyzing the glycosidic bond. Family 4 and 5 UDGs can all remove dU from RNA backbones, suggesting that the riboses on the backbone have little effect on dU recognition and the hydrolysis of C-N glycosidic bond. However, the removal of rU is inefficient, even though the rU is contained within the DNA backbone, suggesting that there is a strong steric hindrance from the 2′ hydroxyl of ribose with which the uracil forms a glycosidic bond. Moreover, removal of 2,2′-anhydro uridine (ahU), hypoxanthine (dI), and 7-deazaxanthine (7-C-dX) is undetectable. Combining our biochemical results and phylogenic trees with other works, we discuss the UDGs’ catalytic mechanism and possible repair reactions of deaminated bases in vivo.

## 2. Materials and Methods

### 2.1. Materials

KOD DNA polymerase (plus+) was purchased from Toyobo (Shanghai, China). Expression vectors, *E. coli* strain BL21 (DE3), and Ni-NTA His•Bind^®^ Resin were purchased from EMD Biosciences (Madison, WI, USA). Oligonucleotides (Supplementary Tables S1 and S2) were synthesized by TaKaRa (Dalian, China). *S. acidocaldarius* DSM639 strain was provided by Professor Sonja-Verena Albers. All other chemicals and reagents were of analytical grade.

### 2.2. Expression and Purification of Recombinant UDGs

The *udgIV* (Saci_0159) and *udgV* (Saci_1756) genes were amplified from *S. acidocaldarius* genomic DNA through PCR using their forward primers and reverse primers (Supplementary Table S2). The amplified DNA fragments were inserted into the pDEST17 vector according to our previous method [31], generating expression plasmids pDEST17-*sacudgIV* and pDEST17-*sacudgV*. The N-terminals (46 residues) of SacUDGV were deleted to prepare the truncated *S. acidocaldarius* UDGV enzyme SacUDGV_Nd. To disrupt the [4Fe-4S] cluster of SacUDGV, two cysteines (cysteine 14 and 17) were mutated to serines by QuikChange Site-Directed Mutagenesis Kit (Stratagene, La Jolla, CA, USA).

Expression and purification of recombinant protein UDGs were performed as described [32]. The *E. coli* Rosetta 2(DE3)pLysS-harboring expression plasmid was used to express the recombinant UDGs via induction with IPTG. Induced bacteria were broken by sonication. The lysate was heated at 65 °C for 30 min before clarifying through centrifugation. The supernatant was used to purify recombinant UDGs through the Ni-NTA His•Bind^®^ Resin column and eluted with a native elution buffer consisting of 20 mM Tris-HCl (pH 8.0), 0.3 M NaCl, 5 mM mercaptoethanol, 200 mM imidazole, and 10% glycerol. The fractions were analyzed by 15% SDS-PAGE. Purified UDG proteins were stored in small aliquots at −20 °C.

### 2.3. Biochemical Characterization of SacUDGs

Oligonucleotides or deoxyoligonucleotides (Supplementary Table S1) carrying an internal dU, rU, ahU, hypoxanthine, and 7-deaza-xanthine were all labeled with a fluorescent assay. The double-stranded substrates were prepared by annealing a group of FAM at the 5′ end, and were used as a substrate in the glycosylase 5′-FAM-labeled oligonucleotides or deoxyoligonucleotides to the unlabeled complementary strands at a mole ratio of 1:1.5. Reactions (20 μL) of UDGIV and UDGV were incubated at 50 °C. After incubation, the reaction was treated with 10 mM (for RNA backbone) or 100 mM (for DNA backbone) NaOH at 90 °C for 10 min and neutralized with HCl. Then, an equal volume of loading buffer (90% formamide, 100 mM EDTA, and 0.2% SDS) was added to the reaction. Reaction products were resolved by 15% 8 M urea denatured PAGE, and then visualized by Phosphorimager (Typhoon 9500, GE Healthcare Life Sciences, Piscataway, NJ, USA). The reaction buffer was optimized for the pH values; NaCl concentration based on the standard reaction buffer contained 20 mM Tris-HCl (pH 8.0), 50 mM NaCl, 1 mM EDTA, 1 mM DTT, and 100 ng/μL bovine serum albumin (BSA). Following optimization, all reactions were performed in an optimal assay buffer containing 20 mM Tris-HCl (pH 8.0), 50 mM NaCl, 1 mM EDTA, 1 mM DTT, and 100 ng/μL BSA.

### 2.4. Multialignment and Phylogenetic Analysis

Clustal Omega (http://www.ebi.ac.uk/Tools/msa/clustalo/) and EXPript3.x (http://espript.ibcp.fr/ESPript/cgi-bin/ESPript.cgi) were used to align each typical UDG selected from the five families of UDG superfamily [33].

## 3. Results

### 3.1. Family 4 UDG Is More Widely Distributed than Family 5

**Figure 2:**
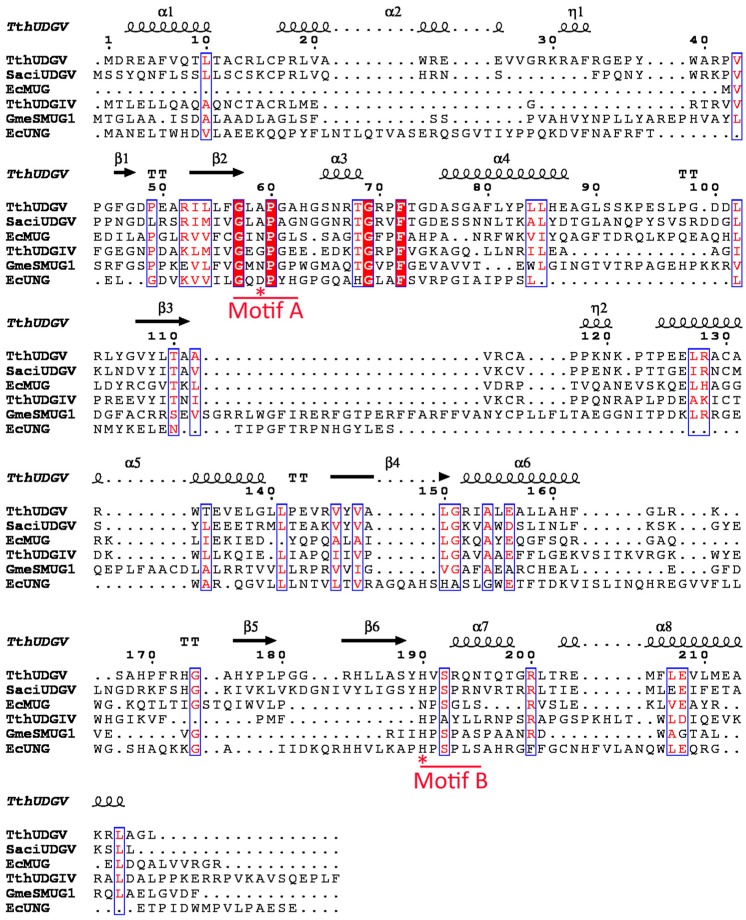
Structure-based sequence alignment of the UDG superfamily. Completely conserved residues among family 1–5 UDGs are boxed with solid lines. The location of the secondary structure elements of TthUDGV is represented at the top of sequences. The conserved motifs A and B are underlined by red lines, and the active residues are marked by red stars. Abbreviations: EcUNG, *E. coli* UNG; EcMUG, *E. coli* MUG; GmeSMUG, *Geobacter metallireducens* SMUG; TthUDGIV, *T. thermophilus* UDGIV; TthUDGV, *T. thermophilus* UDGV; and SacUDGV, *S. acidocaldarius* UDGV. The sequences of the five families’ UDGs were mutialigned using ClustalW and EXPript3.x.

Previous studies showed that family 1–5 UDGs in UDG superfamily have different distribution in three kingdoms [10]. In general, family 1, 4, and 5 UDGs are the most common enzymes for dU removal, whereas family 2 and 3 UDGs are the minor ones. Family 1 UDG is the main glycosylase for removing the dU damages in bacteria and eukaryotes; a few exist in archaea, where family 4 and 5 UDGs are the main dU-repairing glycosylases. Aside from UNG, family 4 and 5 are also the main UDGs in bacteria. However, the distribution of family 4/5 UDGs in three kingdoms is treated as the same family in a previous study [10], and the separate distribution of family 4 and 5 UDGs has not yet been characterized in detail. Considering that family 4 and 5 UDGs have low sequence similarity and different conserved active motif A and B (Figure 2), here we give a comprehensive classification of UDGIV/V in completely sequenced archaea and bacteria (Supplementary data S1). To simplify the analysis, only one strain is selected from the class (archaea) or phylum (bacteria) level. In contrast to the widely existing UDGIV, UDGV is less common and seldom exists alone in one organism. Previously it was suggested that family 4 and 5 UDGs only exist in thermophiles, especially in hyperthermophiles [6,10,16,17]. However, with the increasing availability of sequenced genomes, their presence is also confirmed in many mesophilic and even psychrophilic prokaryotes (Supplementary data S1). Furthermore, many bacteria possess both family 1 and 4/5 UDGs. By comparing the peptide sequences, we found that family 7 has a very high similarity to family 4, which possesses a mutated motif A of GEQP known as GEAP [11]. Therefore, it is noteworthy that, in fact, family 7 is a subfamily of family 4 or a family 4 mutant.

### 3.2. Both SacUDGs Are Uracil-DNA Glycosylases

**Figure 3:**
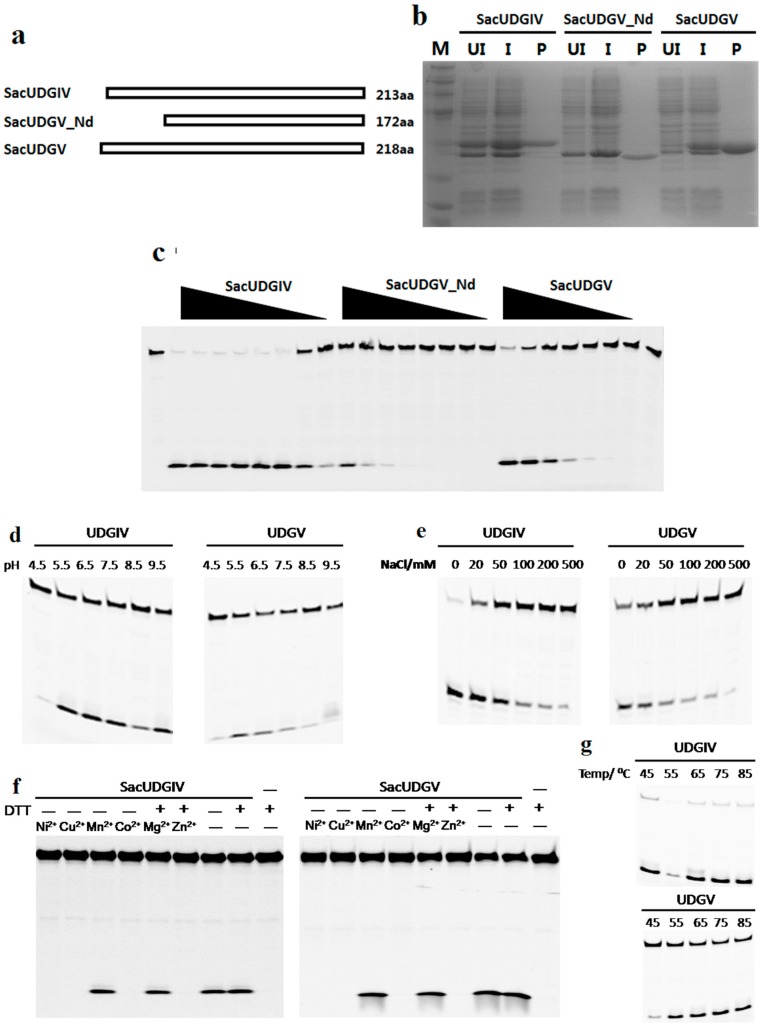
Uracil removal activities of SacUDGs. (**a**) Diagram of three UDGs from *S. acidocaldarius*; (**b**) 15% SDS-PAGE analysis of recombinant SacUDGs recovered from induced *E. coli* extracts. The gel was stained with Coomassie blue R-250. Lane M: molecular weight marker; lane UI: uninduced *E. coli* total proteins; lane I: induced *E. coli* total proteins; lanes P: purified recombinant protein; (**c**) Removal of dU from single-stranded DNA by SacUDGs. Approximately 300 ng SacUDGs were diluted in two-fold and incubated with 0.1 μM ss DNA for 15 min at 50 °C. Effect of pH value (**d**); NaCl concentration (**e**); divalent ions (**f**); and reaction temperature (**g**) on dU removal by SacUDGs. During optimization of reaction conditions about 1 ng SacUDGIV or 10 ng SacUDGV and 0.1 μM dU-carrying single-stranded DNA were incubated at 50 °C for 15 min in assay buffer with various pH value, ion strength, or divalent ions. The reactions were performed at different temperatures for 15 min in an optimal assay buffer.

After purification through native Ni-NTA His•Bind^®^ Resin column chromatography, three UDGs from *S. acidocaldarius*, their domain composition shown in Figure 3a, are shown to be electrophoretically pure, as demonstrated by 15% SDS-PAGE (Figure 3b). Purified SacUDGs, including the N-terminal truncated enzyme SacUDGV_Nd, have strong DNA glycosylase activity on dU damage, resulting in the generation of an apurinic/apyrimidinic (AP) site, which can be cleaved by treatment with hot alkali (Figure 3c).

On confirming the dU removal activity of SacUDGIV and SacUDGV, their respective optimal reaction parameters were determined using single-stranded DNA with internal dU damage (DNA-dU in Supplementary Table S1). Both SacUDGs have high dU cleavage activity at pH values ranging from 5.5 to 9.5 (Figure 3d). A high concentration of NaCl inhibits their enzymatic activity to some extent (Figure 3e). When the concentration of NaCl is higher than 50 mM, the enzymatic activity is decreased by more than 50%. Moreover, divalent ions have different effects on the enzymatic activity (Figure 3f). Mn^2+^ and Mg^2+^ have no clear effect, whereas Ni^2+^, Cu^2+^ Co^2+^, and Zn^2+^ show complete inhibition of the reaction. Meanwhile, the reducer DTT is not necessary even though the two UDGs contain a [4Fe-4S] cluster (Figure 3f, lanes 7 and 8). Both SacUDGs have higher activity at high temperatures ranging from 55 to 85 °C (Figure 3g), and are thermostable proteins (Supplementary Figure S1). The thermostability is consistent with the growth temperature of *S. acidocaldarius*.

### 3.3. The Bases Opposite dU Have Little Effect on the Removal of dU

**Figure 4:**
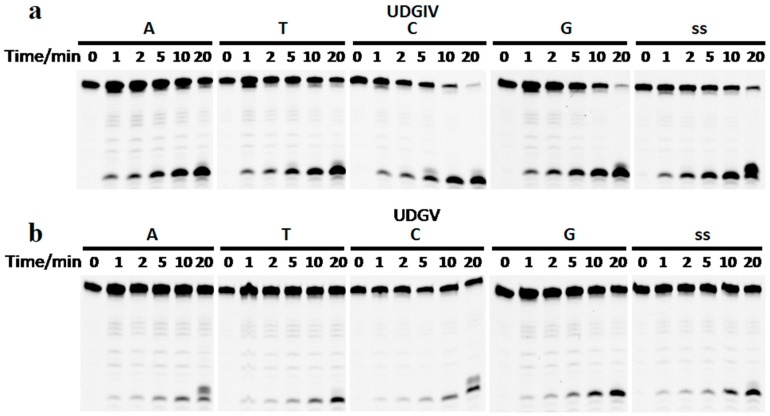
The excision specificity of SacUDGs on double-stranded DNA substrate. Recombinant SacUDGIV (**a**), and SacUDGV (**b**) were incubated with 0.1 μM single-stranded or double-stranded DNA with various bases opposite dU at 50 °C for different times in an optimal assay buffer. The amount of each UDG is 2 ng (**a**) or 10 ng (**b**).

The recombinant SacUDGs can efficiently remove dU from both single-stranded and double-stranded oligonucleotides with different preference. SacUDGIV prefers single-stranded DNA (Figure 4a), whereas SacUDGV prefers double-stranded DNA (Figure 4b). The bases (A, T, C, or G) opposite dU have less effect on the removal of dU in double-stranded oligonucleotides. SacUDGIV removes dU from double-stranded DNA in the following order of efficiency C/U ≈ G/U > T/U > A/U. However, the preference order of double-stranded DNA is G/U > C/U ≈ T/U ≈ A/U for SacUDGV. Compared with bacterial family 5 UDG TthUDGB, the N-terminal of SacUDGV lacks the conserved motif of RKRA that is responsible for binding of complementary strand [25], suggesting that the SacUDGV has a comparable preference for both single-stranded and double-stranded DNAs (Figure 4b).

### 3.4. The Ribose Backbone Decreases dU Removal by UDGs

**Figure 5:**
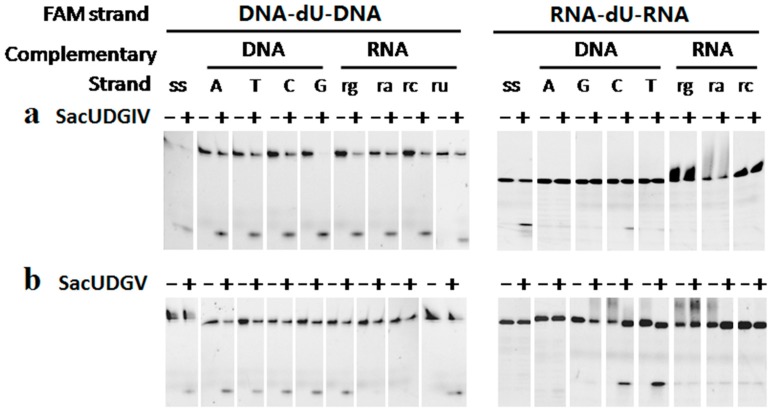
Effect of ribose and deoxyribose backbone on the removal of dU by SacUDGs. SacUDGIV (**a**) and SacUDGV (**b**) were incubated with 0.1 μM single-stranded (DNA-dU-DNA, RNA-dU-RNA) and double-stranded substrates (DNA-dU-DNA/DNA, DNA-dU-DNA/RNA, RNA-dU-RNA/DNA, and RNA-dU-RNA/DNA) at 50 °C for 10 min. After incubation, 10 mM NaOH (final concentration) was added to reaction and heated for 10 min at 95 °C to cleave the AP sites. The amount of each UDG is 2 ng (**a**), and 10 ng (**b**), respectively.

In addition to the interactions responsible for specific recognition of uracil, extensive interactions also exist between UDGs and the backbone of both DNA strands [18,25,34]. As such, we changed the ribose residues in one or both strand and characterized the effect of the backbone on the removal of dU by SacUDGs. All deoxyribose residues were changed to ribose except for the deoxyribose linked with uracil via a glycosidic bond. Results show that two SacUDGs can remove the uracil base from the deoxyribose contained within the RNA backbone, but the change of deoxyriboses to riboses clearly decreases the efficiency of dU removal by UDGs (Figure 5). Two SacUDGs efficiently hydrolyze the glycosidic bond of normal single-stranded and double-stranded DNA-dU-DNA substrates (Figure 5, left panel: lanes 1–10). The complementary RNA strands show clear inhibition of the removal of dU from DNA backbone (Figure 5, left panel: lanes 11–18). However, compared with the dU contained within the DNA backbone, the removal efficiency of dU from the RNA backbone is greatly decreased (Figure 5, right panel). Family 4 and 5 UDGs remove dU from the NA backbone with different efficiency. For SacUDGIV, the complementary DNA strand does not relieve the dU removal from the RNA backbone, and the complementary RNA strands further aggravate the inhibition of dU removal without any detectable product for dU-carrying double-stranded RNA (Figure 5a, right panel: lanes 11–16). For SacUDGV, even if the removal from single-stranded RNA backbone is undetectable (Figure 5b, right panel: lanes 1 and 2), the complementary DNA strand (C and T) improves the removal of dU from RNA backbone to a level comparable to that from the DNA backbone (Figure 5b, right panel: lanes 7–10). Similar to SacUDGIV, SacUDGV does not show any dU removal activity from the double-stranded RNA backbone RNA-dU-RNA/RNA (Figure 5, right panel: lanes 11–16). The time course of removal of a uracil base from the single-stranded substrate RNA-dU-RNA further confirmed the enzymatic activity specific to the uracil linked to a deoxyribose that is buried in a RNA backbone (Supplementary Figure S2).

Although SacUDGs can remove the dU base from the RNA backbone oligonucleotide, the removal of rU from DNA backbone is inefficient (Supplementary Figure S3). This result is also consistent with the low efficiency of hydrolysis of the glycosidic bond between uracil and 2′-modified ribose derivatives, such as 2′-F rU [35]. During catalysis, the 2′ groups of OH and F in ribose impose strong steric hindrance on moving the His residue in motif B during catalysis; as a result, this greatly decreases the hydrolysis efficiency [18,35]. Removal of ahU is also undetectable, indicating that the hydrogen bond between the oxygen atom of 2-carbonyl group and UDG are necessary for recognizing and locating the uracil cycle (Supplementary Figure S3). Unlike other members of family 5 UDG [6], SacUDG_V does not remove the hyperxanthine and xanthine derivative (7-C-dX) buried in DNA (Supplementary Figure S3).

### 3.5. Effects of N-Terminal Sequence on UDGV’s Activity

**Figure 6:**
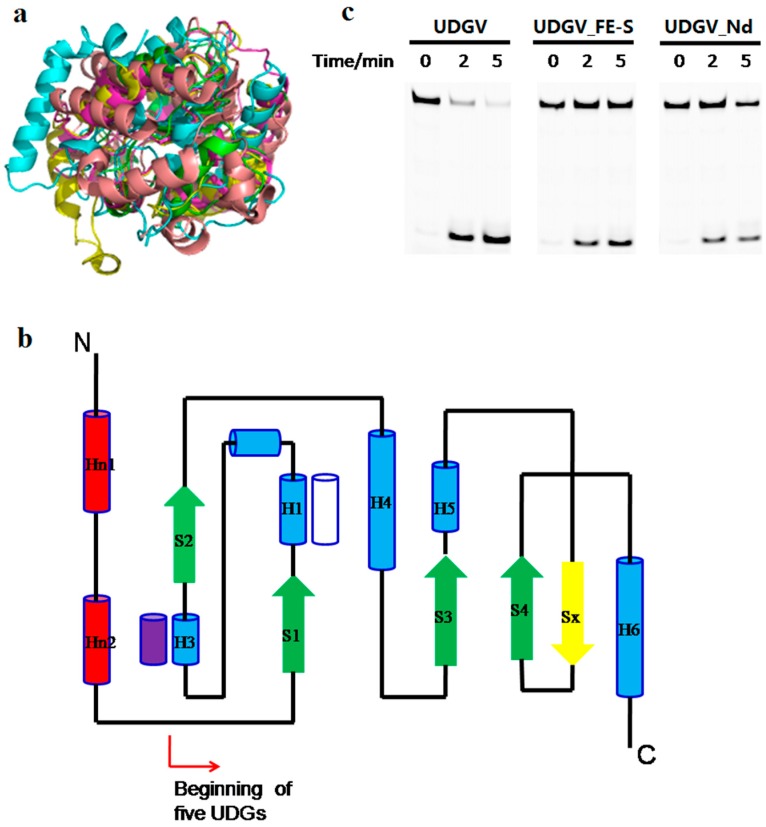
Structural and sequence multialignment of family 1 to 5 UDGs. (**a**) Superimposition of five members from UDG superfamily. Each UDG is shown in yellow (TthUDGV, PDB ID: 2d3y), green (EcMUG, PDB ID: 1mug), cyans (EcUNG, PDB ID: 1eug), wheat (GmeSMUG, PDB ID: 5h98), and purple (TthUDGIV, PDB ID: 1ui0), respectively; (**b**) topology structure of UDG superfamily. The topology of each member of UDG superfamily shows a conserved and unique feature. The secondary-structure elements of the UDG fold are colored as following: common α helices are shown as blue cylinders (the white one is missing in family 1, and the purple one is missing in family 5), and β-strands as green arrows (the yellow one is specific to MUG). The red cylinders are specific to the N-terminal domains of UDGs, excluding the MUG family. (**c**) Effect of N-terminal helices and [4Fe-4S] on dU removal by SacUDGV.

Although the sequences have no clear similarity among the five families of UDG superfamily, each family UDG takes a common structural feature of a four-stranded β-sheet surrounded by α-helices (Figure 6a). Besides the differences in conserved residues for catalysis and damage recognition, there is another clear difference among UDGs: MUG does not have the extra N-terminal topological structure that exists in the other four families (Figure 6b). The function of N-terminal sequence was characterized by deleting it and analyzing the truncated enzyme’s activity. The removal of N-terminal of SacUDGV disrupted one [4Fe-4S] cluster that is responsible for stabilizing the loop for binding substrate [24]. Our results showed that both the truncated enzyme and the [4Fe-4S] cluster disrupted mutants show decreased activity compared with the full-length enzyme (Figure 6c), and the former remains less active than the latter, indicating that both the N-terminal secondary helices and the [4Fe-4S] cluster function in the hydrolysis process. Interestingly, the removal of the N-terminal or mutation of the [4Fe-4S] also clearly decreases the thermostability of SacUDGV (Supplementary Figure S1), suggesting that the [4Fe-4S] cluster is related to the enzyme stability [25].

## 4. Discussion

The members of the UDG superfamily have different substrate preferences (Supplementary Table S3). UNG and UDGIV show the same preference for deaminated cytosine [13,16]. However, UDGs from families 2, 3, 5, and 6 have a stronger activity on two deaminated purine, hypoxanthine and xanthine [6,12,15,36]. Combined with the overall structure conservation and the diversity of binding pockets of damaged bases, we think their similarity in structural folds provides a basis for repairing the deaminated bases by the UDG superfamily, whereas the evolutional diversity of binding pocket decides the recognition and hydrolysis of various specific deaminated bases. Bases C, A, and G all have an exocyclic amino group and thus are susceptible to hydrolysis deamination [37]. dI and dX change the preference of base-pairing, such as dI preferentially pairing with C [38]. Thus, it is beneficial for an organism to have at least one UNG or UDGIV intended for dU removal and one of the other four UDG families for removing deaminated purines.

Family 2 MUG/TDG specifically removes dU from the U/G mismatch, and T from the T/G mismatch that resulted from the deamination of 5-methyl-C paired with G [36,39]. However, the T/G mismatch also comes from the incorrect incorporation of G into template T during DNA replication. In this case, a G:C mutation will be generated if TDG removes the thymine. Hence, it is possible that TDG functions as an enhancer for base mutation.

The family 7 UDG is an inactivated mutant of the family 4 subfamily [11]. This phenomenon is very common, especially in the archaeal Halobacteria class (Supplementary data S1). Halobacteria possess 3 to 4 homologs of family 4 UDG; generally only one protein has the UDG activity and the rest are inactivated. The co-presence of several inactivated UDG and their functions need further investigation.

In minor bacteria (only a few strains from the classes Melainabacteria, Flavobacteria, Bacilli, Clostridia, Alphaproteobacteria, Deltaproteobacteria, and Gammaproteobacteria) and archaea (focused on the classes Methanococci, Methanobacteria, and Methanopyri), no homologs of the UDG superfamily exist in their genome ([10], and see Supplementary data S1 online). Generally, these genomes encode some non-glycosylase genes for repairing the uracil damage, such as endonuclease V [7], endonuclease Q [40], or exonuclease III archaeal homologs [8]. These nucleases exhibit dU-specific endonuclease activity and cleave the phosphodiester bond around the damage; the repair is finished by the other proteins involved in nucleotide incision repair.

Since some prokaryotes do not possess any UDG, it is possible that the dU provides a positive function like that in the higher eukaryotic acquired immunity, where more mutations result from the dU base [41]. Base mutations are beneficial for the adaption of cells under stress conditions. The advantage of more base mutations on the adaption of microorganisms in various environments, which lack dU-repair genes, should be confirmed by evaluating the mutation rate via deep sequencing. Based on the same base pairing property of dU as dT, here we propose a hypothesis for the role of dU during life’s origin and evolution. In the beginning, RNA was the only nucleic acid that served as genetic information and enzyme (ribozyme) molecules. When the mechanisms changed and the modern three-molecule scheme (DNA/RNA/protein) was introduced, the DNA molecule still used uracil, not thymine; both a pyrimidine base and UDGs were absent. Then, the U was replaced by T in DNA, followed by the generation of various UDGs, and it was only used by RNA during transcription. In addition, this hypothesis can be confirmed by constructing a bacterium or archaeon that loses the genes encoding UDGs and dTTP synthetase.

UDGs recognize and extrude the uracil base into a specific extrahelical active site pocket, and then hydrolyze the N-glycosidic bond to release the base. The steric hindrance from 2′ OH of ribose blocks the movement of the larger side-chain of His268 in human UNG and leads to its inability to hydrolyze the glycosidic bond between uracil and ribose [18]. Because the catalytic residue of His is completely conserved and essential for hydrolyzing the glycosidic bond, it is a possible mechanism to discriminate dU from rU by each family of the UDG superfamily.

The annotated gene of *S. acidocaldarius udgV* is shorter in the sequenced genome and lacks the N-terminal peptide (46 residues) that is typically possessed by *udgV* genes of *S. solfataricus* and *S. tokodaii* [30]. SacUDGV_Nd, although with a truncated N-terminal, still has comparable activity to full-length SacUDGV, with approximately 60% residual activity. Since truncation of the N-terminal sequence decreases the enzyme activity, we concluded that the genuine *S. acidocaldarius udgV* gene should contain the upstream 46 amino acid residues, and the N-terminal truncation is just an error coming from a wrong ORF (open reading frame) identification during analysis of the genome sequence of *Sulfolobus acidocaldarius.* Compared with other UDGs, families 2 and 6 lack the N-terminal section, which consists of two α-helices and one β-sheet (Figure 6b). Family 2 MUG shows much lower catalytic activity than the other long family UDGs [14,36]. The decrease in enzyme activity of N-terminal-truncated SacUDGV suggests that MUG might be derived from the long UDGs via an N-terminal truncation.

Various Fe-S clusters function as electron transporters and are essential elements of many oxidoreductases. However, some DNA glycosylases, such as endonuclease III and mutY, have a [4Fe-4S] cluster that is thought to be involved in directly binding the DNA substrate [19,20]. Among the UDG superfamily, family 4, 5, and the TDG HhH subtype also have the [4Fe-4S] clusters. The four cysteines for coordinating [4Fe-4S] cluster are located at the N-terminal of UDGIV and UDGV, but the corresponding functional residues are located at the C-terminal in the TDG HhH subtype, which is similar to HhH type endonuclease III and mutY [19,20,21]. Disruption of the cysteine residues only results in partial activity loss of family 4 UDG [23,24]. Our results also showed that the cysteine residues are not complete essential for removing dU by SacUDGV (Figure 6c), indicating that the [4Fe-4S] of family 4 and 5 UDGs function similarly in stabilizing the enzyme (Supplementary Figure S1), especially the flexible loop anchored by two cysteines [24,25]. However, the Fe-S cluster of endonuclease III is essential for glycosylase and AP lyase via binding DNA substrate [26]. According to the similarity of cysteines between TDG HhH subtype and endonuclease III, the [4Fe-4S] cluster of TDG is possibly the same as that of endonuclease III and different from that of UDGIV and V.

## 5. Conclusions

Two *S. acidocaldarius* UDGs (family 4 and 5) can remove deoxyuracil (dU) base from both DNA and RNA backbones, suggesting that the riboses on the backbone have less effect on the recognition of dU and the hydrolysis of the C-N glycosidic bond. The inability to remove rU from DNA backbone suggests that the strong steric hindrance comes from the 2′ hydroxyl of ribose and prevents the removal of uracil from ribose. Truncation of the 46 N-terminal residues of family 5 SacUDG does not inactivate the enzyme completely, suggesting that the [4Fe-4S] cluster and N-terminal secondary structure are not the key elements in hydrolyzing the glycosidic bond.

## Supplementary Materials

Supplementary Figures S1–S3, Tables S1–S3 and data S1 are available online at www.mdpi.com/2073-4425/8/1/38/s1. Figure S1 Thermostability of SacUDGs and the N-terminal truncated SacUDGV. Figure S2 Time course of SacUDGs on ssRNA backbone carrying a dU. Figure S3 Removal of other deaminated bases by UDGs. Table S1 The oligo(deoxy)nucleotides used for analyzing UDG enzyme activity. Table S2 The oligodeoxynucleotides used for constructing the expression plasmid for UDGs and the mutant enzymes. Table S3 The typical damages processed by each member of UDG superfamily. Supplementary data S1 Phylogenetic analysis of the uracil-DNA glycosylase superfamily.
