# Supplementary material for: Sulfolobus acidocaldarius UDG Can Remove dU from the RNA Backbone: Insight into the Specific Recognition of Uracil Linked with Deoxyribose
Source: Genes (Basel). 2017 Jan 18;8(1):38. doi: 10.3390/genes8010038 (PMC5295032; doi:10.3390/genes8010038)
Supplement: Supplementary file 1 [file genes-08-00038-s001.docx]

Supplementary Materials: *Sulfolobus acidocaldarius* UDG Can Remove dU from the RNA Backbone: Insight into the Specific Recognition of Uracil Linked with Deoxyribose

Gang-Shun Yi, Wei-Wei Wang, Wei-Guo Cao, Feng-Ping Wang and Xi-Peng Liu


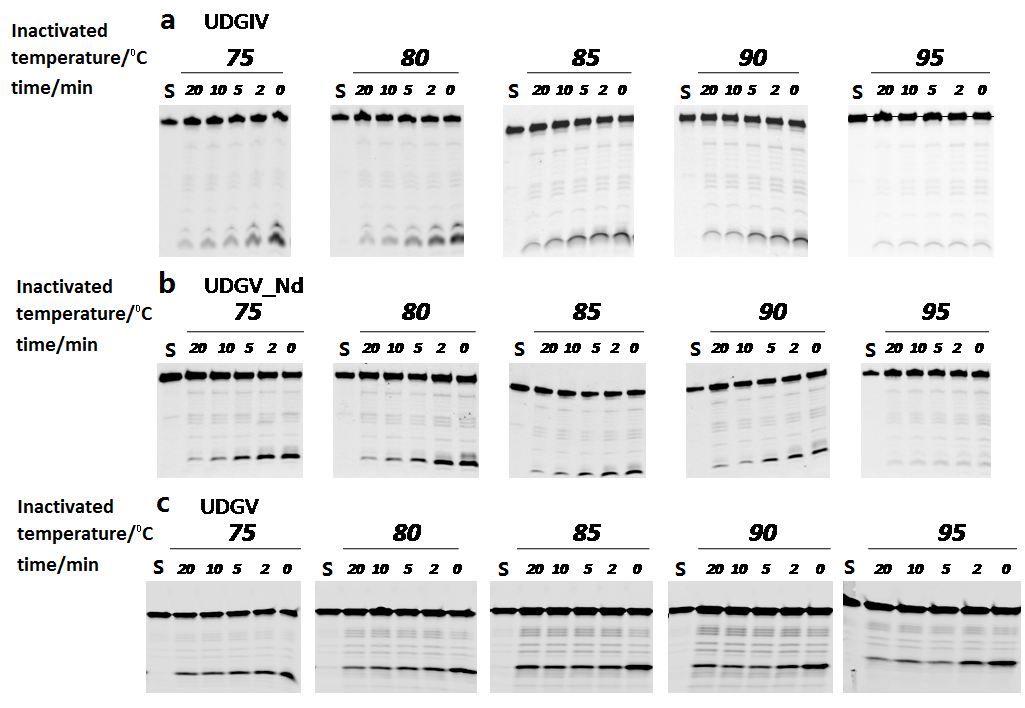


**Figure S1.** Thermostability of SacUDGs and the N-terminal truncated SacUDGV.


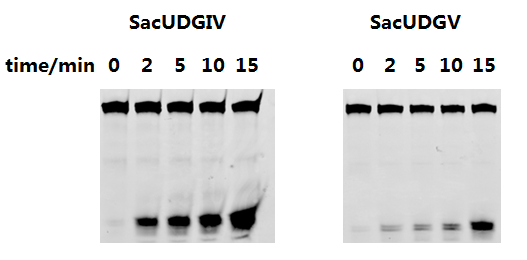


**Figure S2.** Time course of SacUDGs on ssRNA backbone carrying a dU SacUDGIV (5 ng), and SacUDGV (20 ng) were incubated with 0.1 μM ss substrate RNA-dU-RNA at 50 °C for 0, 2, 5, or 15 min.


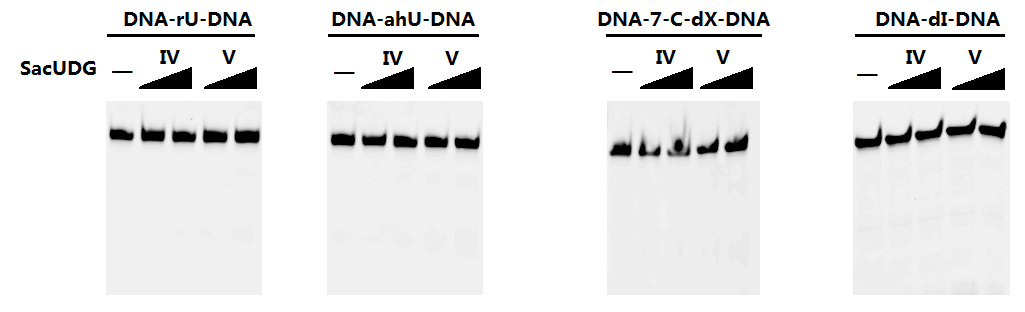


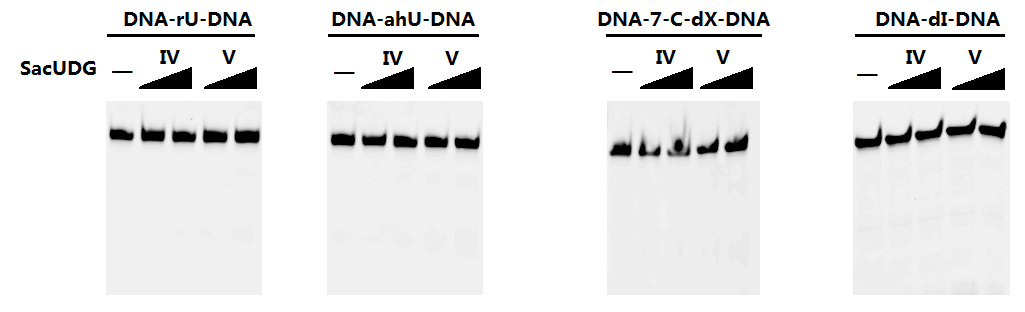


**Figure S3.** Removal of other deaminated bases by UDGs. SacUDGIV (2, 10 g), and SacUDGV (10, 50 ng) were incubated with 0.1 μM ss substrates of DNA-rU-DNA, DNA-dI-DNA, DNA-7-C-dX-DNA, and DNA-ahU-DNA at 50 °C for 15 min.

**Table S1.** The oligo(deoxy)nucleotides used for analyzing UDG enzyme activity.

| **Names** | **Sequences (5′-3′)** | **Damages** | **Comments** |
| --- | --- | --- | --- |
| DNA-dU | *CAGCCAGGTGTCTCACTXAGCCGACTCGCCACAGT | X = dU | 5′-FAM  labeled  strand |
| DNA-rU | *CAGCCAGGTGTCTCACTXAGCCGACTCGCCACAGT | X = rU |  |
| DNA-ahU | *CAGCCAGGTGTCTCACTXAGCCGACTCGCCACAGT | X-ahU |  |
| DNA-dI | *CAGCCAGGTGTCTCACTXAGCCGACTCGCCACAGT | X = dI |  |
| DNA-7-C-dX | *CAGCCAGGTGTCTCACTXAGCCGACTCGCCACAGT | X = 7-C-dX |  |
| RNA-dU | *gucucacuXagccgacu | X = dU |  |
| DNA-c-A | ACTGTGGCGAGTCGGCTAAGTGAGACACCTGGCTG | DNA-dU-DNA  backbone complementary strand | Comple-  mentary DNA strand |
| DNA-c-T | ACTGTGGCGAGTCGGCTTAGTGAGACACCTGGCTG |  |  |
| DNA-c-C | ACTGTGGCGAGTCGGCTCAGTGAGACACCTGGCTG |  |  |
| DNA-c-G | ACTGTGGCGAGTCGGCTGAGTGAGACACCTGGCTG |  |  |
| DNA-c-a | uggcgagucggcuaagugagacaccug |  | Comple-  mentary RNA strand |
| DNA-c-u | uggcgagucggcuuagugagacaccug |  |  |
| DNA-c-c | uggcgagucggcucagugagacaccug |  |  |
| DNA-c-g | uggcgagucggcugagugagacaccug |  |  |
| RNA-c-A | TGCTTCCGTCATCTCCGCTCG | RNA-dU-RNA  backbone complementary strand | Comple-  mentary DNA strand |
| RNA-c-G | TGCTTCCGTCGTCTCCGCTCG |  |  |
| RNA-c-C | TGCTTCCGTCCTCTCCGCTCG |  |  |
| RNA-c-T | TGCTTCCGTCTTCTCCGCTCG |  |  |
| RNA-c-g | ugcuuccgucgucuccgcucg |  | Comple-  mentary RNA strand |
| RNA-c-a | ugcuuccgucaucuccgcucg |  |  |
| RNA-c-c | ugcuuccguccucuccgcucg |  |  |

The DNA bases are denoted by upper-case letters, and RNA bases are denoted by lower-case ones. The asterisks denote the fluorescein group 6-FAM.

**Table S2.** The oligodeoxynucleotides used for constructing the expression plasmid for UDGs and the mutant enzymes.

| **Names** | **Sequences (5′-3′)** | **Genes** |
| --- | --- | --- |
| udg4-f | 5′GGCTCC CATatggagttag acgagatag | SacUDGIV |
| udg4-r | 5′GGATCC GAATTCttaccttttattcctagatc |  |
| udg5-f | 5′GGCTCC CATATG tctagttat caaaattt | SacUDGV |
| udg5-r | 5′GGATCC GAATTCttataacaatgacttggca |  |
| udg5-N47-f | 5′GGCTCC CATAtgattgtcg gcttggcgc | SacUDGV-Nd |
| udg5-C14S+C17S-f | caagtctgctatccAgCagtaagAgccccaggttagta | SacUDGV [4Fe-4S] cluster mutant |
| udg5-C14S+C17S-r | tactaacctggg gcTcttactGcTggatagcagacttg |  |

**Table S3.** The typical damage processed by each member of the UDG superfamily.

| **Family** | **Deaminated Cytosine and Derivatives** | **Deaminated Adenosine and Derivatives** | **Deaminated Guanine and Derivatives** | **References** |
| --- | --- | --- | --- | --- |
| UNG | U, | / | / | [1,2] |
| MUG | U-G,T-G | / | Xanthine | [3,4] |
| SMUG | U | hypoxanthine | Xanthine | [5] |
| UDGIV | U | / | / | [6] |
| UDGV | U | Hypoxanthine | Xanthine | [7,8] |
| UDGVI | / | Hypoxanthine | Xanthine | [9] |

References

1. Lucas-Lledó, J.I.; Maddamsetti, R.; Lynch, M. Phylogenomic analysis of the uracil-DNA glycosylase superfamily. *Mol. Biol. Evol.* **2011**, *28*, 1307–1317.
2. Pearl, L.H. Structure and function in the uracil-DNA glycosylase superfamily. *Mutat. Res.* **2000**, *460*, 165–181.
3. Lee, H.W.; Brice, A.R.; Wright, C.B.; Dominy, B.N.; Cao, W. Identification of Escherichia coli mismatch-specific uracil DNA glycosylase as a robust xanthine DNA glycosylase. *J. Biol. Chem.* **2010**, *285*, 41483–41490.
4. Lee, D.H.; Liu, Y.; Lee, H.W.; Xia, B.; Brice, A.R.; Park, S.H.; Balduf, H.; Dominy, B.N.; Cao, W. Structural determinant in the uracil DNA glycosylase superfamily for the removal of uracil from adenine/uracil base pairs. *Nucleic Acids Res.* **2015**, *43*, 1081–1089.
5. Mi, R.; Dong, L.; Kaulgud, T.; Hackett, K.W.; Dominy, B.N.; Cao, W. Insights from xanthine and uracil DNA glycosylase activities of bacterial and human SMUG1: Switching SMUG1 to UDG. *J. Mol. Biol.* **2009**, *385*, 761–778.
6. Starkuviene, V.; Fritz, H.J. A novel type of uracil-DNA glycosylase mediating repair of hydrolytic DNA damage in the extremely thermophilic eubacterium Thermus thermophilus. *Nucleic Acids Res.* **2002**, *30*, 2097–2102.
7. Sartori, A.A.; Fitz-Gibbon, S.; Yang, H.; Miller, J.H.; Jiricny, J. **A novel uracil-DNA glycosylase with broad substrate specificity and an unusual active site.** *EMBO J.* **2002**, *21*, 3182–3191.
8. Xia, B.; Liu, Y.; Li, W.; Brice, A.R.; Dominy, B.N.; Cao, W. Specificity and catalytic mechanism in family 5 uracil DNA glycosylase. *J. Biol. Chem.* **2014**, *289*, 18413–18426.
9. Lee, H.W.; Dominy, B.N.; Cao, W. New family of deamination repair enzymes in uracil-DNA glycosylase superfamily. *J. Biol. Chem.* **2011**, *286*, 31282–31287.
